# The unmet needs of family members of patients with progressive neurological disease in the Czech Republic

**DOI:** 10.1371/journal.pone.0214395

**Published:** 2019-03-25

**Authors:** Radka Bužgová, Radka Kozáková, Lubica Juríčková

**Affiliations:** Department of Nursing and Midwifery, Faculty of medicine, University of Ostrava, Ostrava, Czech Republic; Institute of Mental Health, SINGAPORE

## Abstract

**Background:**

Caring for patients with a progressive neurological disease (PND) causes stress that may impact on the state of health as well as the quality of life of the caring family.

**Objective:**

The aim of the study was to explore the unmet needs of the family members of patients with PND in advanced stages.

**Methods:**

Grounded theory (constructivist approach) was used to conceptualize the patterns of unmet care needs. Data collection methodology involved focus groups (n = 4) and interviews, in which a total of 52 people participated (patients, family members, and professionals).

**Results:**

Based on the data analysis, three domains (family situation, role of the caregiver, and professional help) were identified, which illustrate the unmet needs. In particular, lack of information about the disease and available support available resulted in a deterioration mutual understanding between the patient, family, and the medical staff; also increased stress for the caregiver, and lowered quality of life for the caring family.

**Conclusion:**

Family members expect health workers to provide them with support, which includes informing them about the possible help available from the health and social welfare systems.

## Introduction

The symptoms of any progressive neurological disease (PND), e.g. Parkinson’s disease (PD), motor neuron disease (MND), Huntington’s disease (HD), or multiple sclerosis (MS), gradually result in full dependence of the patient on the care of others, most commonly family members. Patients have to cope with many changes and losses [1] during their illness, which also affect their family members. Family is a significant part of the social microstructure which providing care and protection to its members especially in times when they are unable to look after themselves. Care for individuals with PND may lead to a range of negative physical, psychological, social, and financial consequences for the caregivers, which may then challenge their ability to continue providing the care. Often, the role of the caregiver is relatively undemanding in the early stages of the disease. As the disease progresses, however, the burden usually grows heavier, and the patients increasingly rely on the caregiver and his or their support in everyday activities. The caregivers may face increased fears and insecurity related to their future; they may experience feelings of guilt, sadness, and frustration, negative changes in their lifestyle (including limits to work and social activities), as well as a deteriorating financial situation, especially due to the loss of income [2]. In a systematic review, Aoun et al. [3] discuss the burden on family members arising from the lack of time they have for themselves. Giordano et al. [4] identify high anxiety and low economic status of carers as burden predictors. This, all in turn, may result in significant psychosocial consequences, including lowered quality of life, emotional and financial stress, fatigue, sleep disorders, social isolation, and aggravated risk of neuropsychiatric symptoms and chronic diseases [5]. Researchers describe how care for a partner or another family member with PND leads to stress, psychosocial distress, and it lowers the quality of life of the caregivers [6–8]. For this reason, examining the unmet needs of family members is important, as making adequate provision for the needs of family caregivers by health and social service providers can reduce the burden of care upon them. Higginson et al. [9] state that needs assessment is part of the process of developing services; and its findings should be adopted and implemented from the outset.

Borreani et al. [10] conducted research into the unmet needs of family members taking care of patients with MS. They emphasized the pressing need for qualified personnel and care coordinators in day-to-day home care. Bowen et al. [11] point out that family members are often not even sure of the type of support they require. Auon et al. [12] identified more unmet needs in the emotional and spiritual domains of caregivers of patients with PND than in caregivers of patients with other diagnoses.

In the Czech Republic (CZ), the attention paid to informal caregivers (especially the families of dying patients) and their needs, is inadequate. Mostly it is the patient who is in the spotlight, but the needs of the family members are often overlooked, despite the fact that providing care at home causes them various physical, psychological, social, and financial problems. Likewise, research tends to deal more with the unmet needs of patients with PND themselves [13–17], while carers currently receive little consideration [18]. Although Golla et al. [13] also focus on the unmet needs of patients with MS, they do mention a group of healthcare professionals interested in the unmet needs of relatives. A number of authors [19] note that even when research does focus on the unmet needs of caregivers, the family members involved tend to put the unmet needs of their care recipients together with their own, rarely focusing exclusively on their own situation.

Discussions about connecting palliative and neurological care in patients with PND are increasingly common nowadays [7, 20–22]. Research is also focusing on palliative care needs [23–26] in order to provide adequate palliative care services for patients in an advanced stage of PND, and their caring family members. The approach connecting rehabilitation, with neurological and palliative care, with the aim of optimizing care management for PND patients and their family caregivers is known as “Neuropalliative Rehabilitation” [27], and is recommended in the early stages of the disease, before the development of cognitive deficits. The concept of Neuropalliative rehabilitation involves “a holistic approach to the care of neurological patients with significant disability, complex needs and a potentially shortened life-span. It is patient-centred and involves diagnosis of clinical problems at all stages, rehabilitation to maintain function, care coordination and appropriate palliation to relieve symptoms” [28,29]. Care is multidisciplinary, and focuses also on support for family members.

The aim of our research was to explore the unmet needs of family members of patients with progressive neurological disease (e.g. multiple sclerosis, neurodegenerative diseases–PD, HD, and motor neuron disease) in advanced stages of the illness in connection with quality of life, and to develop a substantive theory of unmet needs of family members of patients with PND.

The following specific research questions were established: a) *What are the unmet needs of family members of patients with PND*? *How do participants describe them*? b) *What is the impact of caregiving on quality of life of caregivers*, *and their physical and mental health*? *How do they talk about it*? c) *What problems and unmet needs do family members have regarding health and social care support services*? *What problems do they emphasize*?

## Methods

Grounded theory (constructivist approach) was used to conceptualize the patterns of unmet care needs [30]. Qualitative research is “any type of research that produces findings not obtained by statistical procedures or other means of quantification”. Grounded theory is a methodology that seeks to construct theories about issues of importance in peoples' lives [31]. Constructivist grounded theory is a popular method for research studies, particularly in the disciplines of psychology, education, and nursing [32], and is attributed to Kathy Charmaz. It adheres to a constructivist philosophical approach, wherein both the researcher and participants mutually co-construct meaning during data collection and analysis [30]. Constructivist grounded theory reshapes the interaction between researcher and participants in the research process, and, in doing so, brings to the fore the notion of the researcher as author. Constructivist qualitative methods mean entering research participants' worlds. Data do not provide a window on reality. Rather, the ‘discovered’ reality arises from the interactive process and its temporal, cultural, and structural contexts [33], with an emphasis on keeping the researcher close to the participants through keeping their words intact in the process of analysis [32].

### Sample

A total of 52 study participants participated in the research, included in the folloving three groups:

1. Outpatients with PND (n = 11; six men, five women; age range 36–82 years),
2. Family members of patients with PND (n = 6; five women, one man; age range 42–69 years),
3. Professionals working with the patients with PND (n = 35; six doctors, 16 nurses, three physiotherapists, two occupational therapists, four social workers, two psychologists, and two hospital chaplains; age range 25–68).

The selection of the participants was intentional, and based on the stated criteria: 1) a patient, or family member of a patient with a specific neurological disease (multiple sclerosis, Parkinson’s disease, atypical parkinsonism, Huntington’s disease, motor neuron disease), at least one year after the diagnosis was made, age >18 years, MMSE ≥ 24 points, informed consent in writing; 2) professionals–professional qualification for the given position, at least one year´s experience of providing care to patients with neurological disease.

Professionals, family members, and patients were contacted in all regions of the Czech Republic. The method of obtaining the sample was the snow ball technique. First patients, family members, and healthcare professionals were contacted in the therapeutic center for demyelinating diseases, and the outpatient department for extrapyramidal diseases and cognitive disorders of the neurological clinic at University Hospital Ostrava. The participants who met the inclusion criteria were contacted by the study coordinator (RK), based on recommendations.by the head of the center. The remainder of the participants were selected based on recommendations from the studied individuals, and were also contacted by the study coordinator. Emphasis was placed on participant´s experience of the specificed topic. Participants were accepted until theoretical satiation of the sample was achieved (theoretical sampling).

### Data collection

The methods of data collection included individual open-ended audio-taped interviews and focus groups–ten interviews with patients (four with severe MS, six with severe Parkinson’s syndrome PS); six interviews with family members of patients (two with MS, one with PS, one with Huntington’s disease HD, two with NMN); three interviews with professionals (a doctor, a nurse, and a social worker); four focus groups (in total 31 health and social care professionals, and two hospital chaplains). No more than three family members were caring for the patients included in the study at any given time.

Focus groups and individual interviews are methods that generate qualitative data that include experiences and insightful perspective beyond that typically detected in quantitative analysis [34].

The individual interviews took place in a suitable room with no-one else present. When the purpose of the study was explained and the participant’s consent obtained, the interviewer asked questions focusing on: a) problems of family members during care, b) problems in family relationships, c) the impact of caring on physical and mental health of family members, d) problems with professional help, and e) planning end of life care. The participants talked freely about the topic. Afterwards, the interviewer asked additional questions related to the responses. The individual interviews lasted 30 to 70 minutes, with a median time of 56 minutes. The focus groups discussed the experiences of the professionals of the given issue. The following main questions were asked: *“Based on your experience*, *what do you think are the needs and most frequent problems of family members of patients in advanced stages of neurological disease*? *What support is given to the caregivers in the current system of care and what support do they need*?*”* The average duration of the focus groups was 120 minutes. All interviews and focus groups were recorded on a voice recorder, and subsequently transcribed verbatim. The data collection formed part of a qualitative study for the research project run by the Czech Ministry of Health (no. 17–29447) entitled “Neuro-palliative and Rehabilitation Approach to Maintaining Quality of Life of Patients in Advanced Stages of Specific Neurological Diseases”.

### Data analysis

Data analysis occurred as data were collected. Theoretical sampling ceased when theoretical saturation occurred. Theoretical saturation was considered to be achieved when the identified themes were robust, and no new codes emerged from the data. The quality and sufficiency of the data, and the level of thematic saturation were assessed throught a critical realist lens, and evaluated as to whether sufficient background data about people, processes, and settings were available to allow researches to understand and portray the full range of the context of the study, as well as to identify causal mechanism and facilitators of collaboration [30].

The discussions and interviews were recorded, transcribed verbatim and anonymized to ensure confidentiality. Grounded theory coding includes at least two main phases: 1) an initial phase involving the naming of each word, line, or segment of data; 2) a focused, selective phase that uses the most significant or frequent initial codes to sort, synthesize, integrate, and organize large amounts of data [31]. To enable interpretation, conceptualization, and re-integration of the data, the open, axial, and selective coding were used. Two researchers (RB, RK) independently codified the raw printed transcript, without the use of software. The researchers conferred regularly to discuss coding, analysis and data interpretation. During open coding, various terms were created. As the coding unit, a semantic unit was selected and, a line by line, of the transcribed interviews were coded. After the researchers discussed their interpretations, a coding frame was created. Subsequently, the codes were categorized by means of the constant comparative method, and were then named. In addition, the characteristics of the codes, and the dimensions of these characteristics were recorded.

A third type of coding is axial coding, to relate categories to subcategories. Axial coding specifies the properties and dimensions of a category [30]. During axial coding, we proceeded to re-order the data after the open coding by creating links between the subcategories and categories [32]. When the main categories were obtained, a third selective coding was performed, and domains (the highest level of abstraction) were determined. When engaged in axial coding, Strauss and Corbin [31] apply a set of scientific terms to make links between visible categories. We used an organizing scheme based on that of Strauss and Corbin: 1) conditions, circumstances, or situations that form the structure of the studied phenomena (length and progression of disease of a loved one); 2) actions/interactions, participants' routine or strategic responses to issues, events, or problems (three constructs of unmet needs–situation in the family, role of caregiver, professional help); and 3) consequences, outcomes of actions/interactions (decreasing QoL, increasing caregiver burden). The data analysis aimed to identify the central category of the studied material (unmet needs), which became the center of the hierarchical system of categories.

The study met COREQ criteria for reporting of qualitative research [35].

### Ethical aspects

The study conforms to the provisions of the Declaration of Helsinki, and was approved by the ethics committees of University Hospital Ostrava (no. 486/2016). Patients and family members were properly informed about the purpose of the study, and gave written informed consent to participation in the study.

## Results

**Fig 1:**
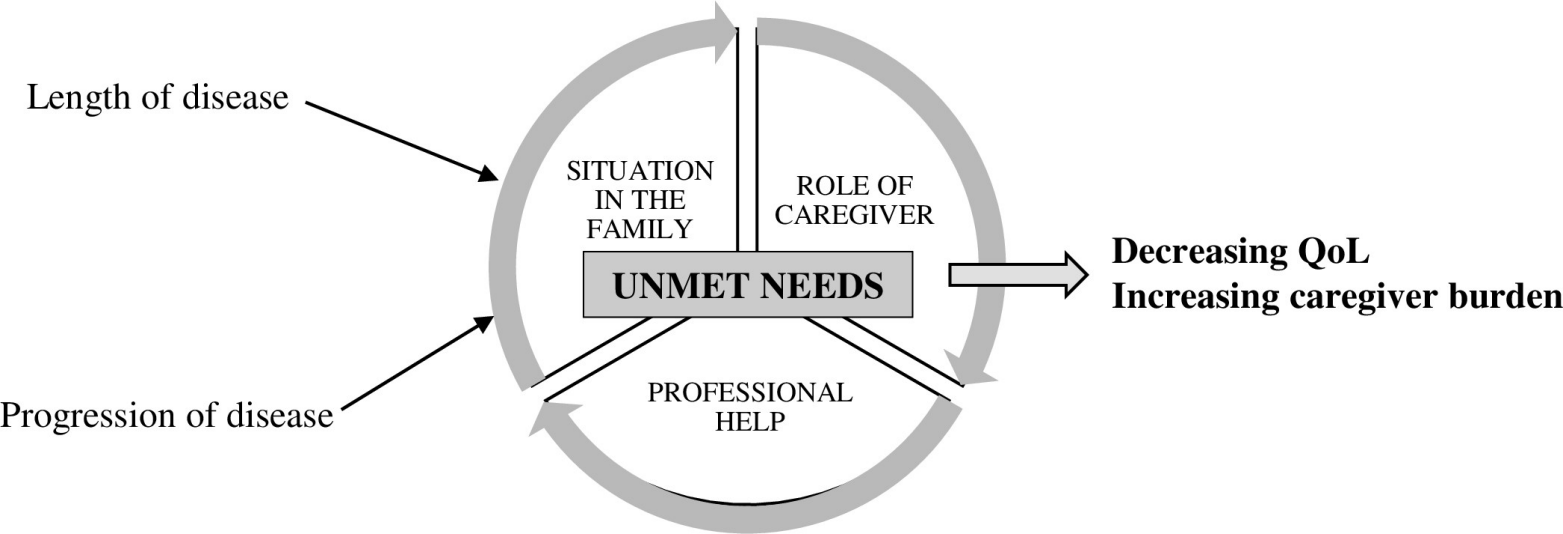
Final model of data analysis.

The length and progression of disease of loved ones with PND resulted in the unmet needs of family members described. Based on the data analysis, three main categories were determined (see Fig 1): 1) situation in the family, 2) the role of the caregiver, and 3) professional help. The unmet needs in all constructs decreased quality of life of family members and increased the care burden.

An overview of the categories (constructs) and subcategories (themes) summarizing the areas of unmet needs are presented in Table 1. Each category (construct) and subcategory (theme) is discussed in separate sub-sections below. Selected quotations are used to illustrate the data (Tables 2–4). Participants are identified by the following letters: P (patients), FM (family members) and, HSCP (health and social care professionals).

**Table 1:** Overview of established categories and subcategories.

Domaine	Category	Subcategory	Participants*
Situation in the family	Lack of understanding in family	Communication in family	FM
		Comprehending the patient’s state of health	P, FM, HSCP
	Demands of psychological wellbeing	Suppressing own emotions and needs	FM
		Psychological dependence	FM
	Worries of burdening the FM	Worries of burdening the direct caregivers	P, FM, HSCP
		Worries of burdening the extended family	P, FM, HSCP
Role of caregiver	Status of caregiver	Lack of recognition in society	P, FM, HSCP
		Problems at work and after returning	FM, HSCP
	High demands on the caring family	Restrictions in personal activities	FM, HSCP
		Limited contact with other family members	FM
		Worries about managing long-term care	FM, HSCP
		Physical exhaustion	P, FM, HSCP
		Psychological exhaustion	FM, HSCP
		Family breakup	HSCP
Professional help	Lack of information	About diagnosis and disease	FM, HSCP
		About the role of caregiver	HSCP
		About nursing services	HSCP
		About manipulation of patient	FM, HSCP
		About financial support	HSCP
	Insufficient support of family	Support by health workers	FM, HSCP
		Accepting the patient’s state of health	HSCP
		Support by social workers	FM, HSCP
	Problems when securing health care	Seeing the neurologist	FM
	Problems when securing home care	Readiness for the role of caregiver	FM, HSCP
		Violating privacy	FM, HSCP
		Affecting the daily regime	FM
	Problems when providing care at the end of life	Discussion about the plan of the end of life	FM, HSCP
		Intervention at the end of life	HSCP
		Support when accompanying the dying person	HSCP
		Support after death	HSCP

*P–patient, FM–family members, HSCP–health and social care professionals

**Table 2:** Situation in the family additional supporting quotes.

Subcategories	Quotes
**Lack of understanding in the family**	• *“Many times they live with completely unreasonable notions of what their relative is able to do…”*. (HSCP) • *“She does not read much*, *which quite surprises me… Then*, *she expects us to have some program but she does not want to prepare it for herself at all*. *She should have a more active approach…”*. (FM) • *“The family can’t even see that his state is so bad that it cannot get better*. *Sometimes they are so obstinate that in a manner of speaking*, *they attack the person when care giving*. *And they continue to have expectations…”*. (HSCP) • *“…when the hot season comes I am afraid to let my husband go outside… He says that he can manage*. *That’s why we have quarrels … I fight my fears and my husband feels humiliated*.*”* (FM)
**Demands of psychological wellbeing**	• *“…probably the worst thing for me is the idea that somebody depends only on you*. *When you have little children*, *they will soon grow up and will not need you anymore*, *but knowing that somebody really depends on you is sometimes quite hard… I cannot go somewhere for long*, *I can´t go on holiday for a week…”*. (FM) • *“As soon as I leave*, *the psychological wellbeing of my husband ends and his health state declines…”*. (FM)
**Worries of burdening the family members**	• *“…they don’t want to be any trouble for the family*, *they perceive it as a burden*.*”*(HSCP) • *“I think he doesn’t feel so much guilt any more—that he is such a burden*. *He has the financial support*, *he gets some money that he can give to the caregiver…”*. (HSCP) • *“I can’t ask my son*, *who is 43*, *to come every day*. *He has his own life*, *he has his work*. *He can’t come daily… I don’t want that*. *It wouldn’t be a normal life for him*.*”* (FM)

**Table 3:** Role of caregiver additional supporting quotes.

Subcategories	Quotes
**Status of caregiver**	• *“The status*, *the social status*, *of the caregiver is also lower… It is even lower than the professional caregiver…”*. (HSCP) • *“…then I have no chance to come back to my original profession*.*”* (FM)
**High demands on the caring family**	• *“We must plan our personal program or life*. *We must limit our personal interests and schedule everything with my husband so that someone is always at home…”*. (FM) • *“They from the 24-hour home care … They need some assistant who might be there for a longer time*.*”* (HSCP) • *”In this situation*, *there is not much time and energy to solve or live lives with the next generation*, *with children*, *or to take part in their life*, *or to find your place in life*. *Quite simply*, *there isn´t time…*.*”* (FM) • *“For four years I didn’t sleep*, *or rest*, *because she stopped swallowing*, *she couldn’t eat*, *so basically PEG*. *It meant that for 24 hours I was on alert*. *Every two and a half hours*, *PEG*. *Meanwhile*, *pills that she had to take at regular hours*. *I walked with her*, *exercised with her*. *She had little balls to move her hands*. *We did what we could so that her body worked at least a little*.*”* (FM) • *“At first it was both (physical and psychchological)*, *now it is mostly psychical*. *It is long*, *and it doesn’t progress in any direction*.*”* (FM) • *“I used to visit a family*, *they had a daughter with MS*. *She managed to live with the disease for a very long time and basically she destroyed the whole family around her*. *They all became the victim of her disease*.*”* (HSCP)

**Table 4:** Professional help additional supporting quotes.

Subcategories	Quotes
**Lack of information**	• *“The family members don’t know the seriousness of the diagnosis at all*.*”* (HSCP) • *“…they come totally miserable*, *they don’t know what to do…”* (HSCP) • *“When the family members get their relative home*, *they don’t have any information how to work with that person*. *How to turn him or her around*, *how to change the nappies*.*”* (HSCP) • *“It would help me to go to the rehabilitation with my husband*, *so that I could also learn how I can exercise with Mira at home*, *how to make the tasks easier*, *when I lift him up*, *when I turn him around*, *how to care for him with respect to the physical side*, *and therefore I harm my own body*, *because I don’t know how to do it*, *and then I fight for my own health as well*.*”* (FM) • *“The care staff don’t have the information about the correct approach to caring for a person with PD*.*”* (HSCP) • *“…there is nobody who could coordinate the information*, *who could provide them complete information in some way*.*”* (FM) • *“Lots of troubles could be forestalled if they knew at the right time how to look after the patients…”*. (HSCP)
**Insufficient support of family**	• *“…they are exhausted*, *they can’t manage anymore*. *The support is*, *however*, *quite small*, *or rather negligible*.*”* (HSCP) • *“…in front of the door the tired*, *broken*, *worn out family member is sitting*, *waiting for the ambulance to take the patient back home*, *and he is in it quite alone*. *The emphasis is on the whole situation*, *not to see only the whole person but to see the whole environment of the family*.*”* (HSCP) • *“Our aim is that they begin accepting this state*, *because it is not possible to accept these things*. *The family can’t accept it*, *they will always fight it…*, *but we need to learn to accept it*.*”* (FM) • *“The social workers don’t help much*, *instead they ask the caring person to get something*, *but they don’t tell them where they can get it*. *You should be looking after a sick person who needs attention twenty-four hours a day*, *but at the same time you have to submit lots of stamps and documents in some office and how can you do it in time*?*”* (FM) • *At the social security office*, *even though the disease is there*, *you have to keep going there*, *dealing with papers… In that hallway*, *I went to every single door*, *and they kept sending me to the next door*. *I started shaking and sat down on the bench and I started screaming (laughter)*. *I did that on purpose… I had really had enough of it*.*”* (FM)
**Problems when securing the health care**	• *“We used to go to the neurologist*. *The worst thing for me*, *even worse than the whole disease of my husband*, *was the feeling that we had to go there every three months*. *I took him in the*. *To put him in the car*, *downstairs*, *in the wheelchair*, *to drop him of there—you can’t find anywhere to park there*, *so finally I sit him in the wheelchair*, *leave him there*, *and come back*. *And I never waited less than three hours*, *even though we had an appointment*. *Nobody can imagine how it is with the sick person*, *and now you have to wait there for three hours*.*”* (FM)
**Problems when securing the home care**	• *“When it goes fast the family is not able to cope with the existence of the disease*, *not even the fact that they will have to take care and reorganize their drive to work or child care*.”(HSCP) • *I very often face the situation whereby the patient or the family do not agree with somebody entering their house*, *that some stranger will care for them…”*(HSCP)
**Problems when providing care at the end of life**	• *“We don’t offer it actively*, *we wait until the family or the client come*, *and then we process the order*, *but we don’t initiate it*.*”* (HSCP) • *“…the patient wouldn’t want it but the family puts pressure on the doctor so that he applies it*, *that he uses PEG*, *so that the patient still receives some nutrition*, *since it may improve…”*.(HSCP) • *“A patient with HD had an 18-year old daughter who kept coming to see her mother*. *When the mother reached the last stage*, *the daughter took it very hard psychologically… She always came in tears*. *We had to calm her down…”*. (HSCP) • *“…it is still hard to justify that he will be somewhere where he is not at home*. *Maybe the right for home is anchored in me… But probably I need to get over it*, *taking into account the interest of the family and its survival…”*. (FM)

### Situation in the family

Situation in the family is comprised of three parts: *lack of understanding in the family*, *demands of psychological wellbeing*, *and worries of burdening the family members*.

#### Lack of understanding in the family

Lack of understanding in the family is the first problem/unmet needs in the construct of situation in the family which affecting family members´ quality of life. Particularly during the first stage of the disease, the family situation may be complicated due to insufficient communication between the patient and the family members regarding the difficulties of the sick person. It may be caused by the fact, that the patient (having the role of a parent) does not want to aggravate the situation for their children, and thus does not talk to them about their health problems. In this way, however, the process of preparing for the role of a caregiver is made more difficult: *“…naturally it is the relationship of a parent and children and it depends whether the parent talks about their problems with the children or not… if the person talks about it only with the doctor*, *then the adult child does not know how serious it is*.*”* (FM)

Moreover, the participants (P, FM, HSCP) agreed that misunderstandings and tensions in the family often occur because of the different views of family members and patients regarding the ability of the patients with PND to do various activities. Family members have a lack of information about the disease. Some family members refer to these situations as groundless fears: *“She is scared of some things quite needlessly*. *She does not want to go out with us…”* (FM). Another woman explain how, after some time, she accepted (after receiving more information about the disease) that the ill person was genuinely physically incapable of performing certain activities, and changed her attitude: *“At first*, *we kept persuading her that she had to be active*, *she had to exercise… And we were really bad*, *because we could never fully grasp how she felt and that she really could not do it*, *and that when it was not possible*, *she could not exercise*. *So*, *gradually we stopped doing that*. *Today*, *we simply ask her*, *and when she downright doesn´t want to*, *we don’t try to persuade her…”*. (FM)

Misunderstanding and conflicts may also arise out of excessive worries of the caregiver about the safety of the ill person when away from home.

#### Demands of psychological wellbeing

The link between the psychological and physical state of many patients with PND put great demands on the family. This is the second component of these categories affecting family members´ quality of life. The family members endeavor to create a positive atmosphere in the household in order to prevent further physical decline, and, thus, a greater physical burden, and consequently they often suppress their own needs: *“It´s psychologically demanding when you need to be careful not to bring home any bad mood*, *because then the avalanche-like effect starts*. *At such times*, *my husband’s psychological state worsens*, *his health state declines really fast… and the physical burden follows…”*. (FM)

In addition, family members (especially those of productive age) also often described how they needed to cope with the dependence of their relative on them. The psychological dependence of the sick person on the caregiver often prevents them from doing anything outside the home.

#### Worries of being a burden on family members

The situation in the family, and mutual relationsship among the members differ in various families. Regardless of the intensity of the mutual relationships, many patients did not wish to place any physical burden on their relatives. A patient himself explained it as follows: “*Imagine that you must look after somebody all the time… Even though you like the person … I wouldn´t like it*.*”* (P). Caring spouses did not want to cause stress for other family members due to the difficulties of care. This situation has led to misunderstandings. One form of help are caregiver benefits (financial support), which provide the family with some degree of compensation.

### Role of caregiver

The role of caregiver is comprises of two parts: *status of caregiver and high demands on the caring family*.

#### Status of caregiver

The first problem/unmet needs in the construct of role of caregiver which affects family members´ quality of life is status of caregiver. The participants mentioned insufficient recognition of the role of the caregiver in the society, and, thus, low social status. When caring for a sick person, the social position of the whole family often changes. People of productive age providing care to somebody described problems with performing their work duties and returning to work after the patient had died. Some family caregivers opted for early retirement to be able to provide care to their relative: *“My husband retired early because of me*.*”* (P)

#### High demands on the caring family

Due to the demands of providing care, caring family members had to limit their interests and daily activities. This is the second component of these categories affecting family members´ quality of life. One patient stated that caring limited her husband and his personal life: *“…I am aware that because of me his life is very limited*.*”*(P). 24-hour care is very difficult for the family, when they cannot leave the house. The strenuous nature of care often interferes with the development of relationships with children, and the enjoyment of one’s own life.

Family members described how the burdens of caring exacted a physical and psychological toll. Some family members felt that caring was too strenuous for them and were not sure if they could cope with it for the whole course of the disease: *“I know that I am able to cope with this tempo and strain maybe for ten years*, *and then I will depend on someone else who will help my husband…”*. (FM)

The high demands on family members when providing care can lead to exhaustion: *“I meet families that are burnt out because it was all on the shoulders of certain individuals*, *and they don’t even know where to get help…”*. (HSCP). Even a patient himself perceived the exhaustion of the caring relative during his stay in a rehabilitation facility: *“The main thing is*, *that my wife …* [crying] *… that she will have some rest…”*. (P)

### Professional help

The next construct—“Professional help”–comprises five parts: *Lack of information*, *Insufficient support for family*, *Problems when securing the health care*, *Problems when securing the home care*, *Problems when providing care at the end of life*.

#### Lack of information

Lack of information is the first problem/unmet needs in the construct of Professional help which increases the burden on family members. The participants agreed that the most common unmet need of the family is lack of information, both regarding the disease and other care options. The follow-up care workers also encountered lack of information among family members: *“…we discover that the information that the caring person has is zero…”*. (HSCP)

Lack of information was apparent even at the time of diagnosis, when there was a sudden change in the family, especially when the first symptoms of the disease require care for the patient: *“…when this situation happens it usually comes quite suddenly*. *The caregiver is not ready for that*, *doesn’t know what to expect*, *what it entails*.*”*(HSCP)

Lack of information was also described by the participants (FM, HSCP) also in connection with the home nursing service. They do not have much information about basic care, such as prevention of decubitus, difficulties with eating, bowel movements, and incontinence. Often they do not know the tools available. This was confirmed by a physiotherapist with respect to manipulating a patient: *“The families don’t know how to touch him*, *what they can and cannot do with him physically…”*. (HSCP).

Even direct care workers in the facilities of social welfare and health services do not have sufficient information. Therefore, they are not able to properly educate family members.

Ignorance of the seriousness of the disease in its advanced stages resulting in unreal expectations of family members of an improvements in the state of health was also apparent: *“The family members don’t know how serious the diagnosis is*, *and it raise hopes in the patient that in the nursing home they will get them back on their feet*, *they will help*, *even though the person is in a very serious advanced stage of the disease*. *Thus they nourish the hopes of the sick person that their state will get better*.*”*(HSCP). The follow-up care workers also experience these situations: *“…they expect his state to improve…”*. (HSCP)

Lack of information was also discovered in the area of financial support, and the system of social benefits, along with a lack of coordination in the information provided. All participants (FM, HSCP) agreed that adequate information would prevent many problems related to caring.

#### Insufficient support of family

The tiredness of the family is often related to insufficient support from community services. The medical staff emphasized that, particularly in the advanced stage of disease, families often have greater need of support than the patient: *“They come with him full of despair… Often the caring person needs the support more than the patient*.*”* (HSCP). Frequently, in spite of this, only the patient is provided with care in medical facilities, while families do not receive sufficient attention.

Family members felt in great need of support when having to accept the reality of the disease of their close relative, support which, usually, they are not provided with: *“We need to accept that someone who*, *for instance*, *maintained them until now or normally worked*, *now apathetic*, *with decubitus*, *defecating under himself or herself…”*(FM). The family should be provided with psychological support, especially when the patient´s the state deteriorates.

Family members also described insufficient support with respect to securing social help, especially all day care for the ill person. The inadequate support provided by the social workers can result in aggressive behavior from family caregivers.

#### Problems when securing the health care

Family members talked about the problems they experienced securing healthcare, mentioning regular checkups with the neurologist, difficult journeys to medical facilities, and in particular, long waiting times.

#### Problems securing the home care

When securing home care, there is no preparation of the family members for the role of caregiver, which should begin as soon as the diagnosis is given. Home care is usually secured when the health state of the patient, and their self-reliance significantly deteriorate. Family members often do not have enough time to decide whether they will provide the care, particularly when the health state worsens suddenly. Family members also mentioned another issue when deciding about securing home care–the fear of home care provided by a stranger: *“I realized that actually I am scared*. *I am scared to let anyone in the apartment… whether I can trust that person…”* (FM). An additional problem when securing the home care is the necessity of adjusting the daily schedule of the family and the patient according to time the given service is available: *“…I wanted to hire some paid service but my husband doesn’t want to go to bed at 5 pm*. *But they finish at 5 pm…”*. (FM)

#### Problems when providing care at the end of life

Families are not usually offered any consultation over plans for the end of life, unless the family specifically requests it. At the same time, the health workers agreed that family members should have the opportunity to discuss the options for care at the end of life at a time when the patient does not have any cognitive deficiencies.

By providing sufficient and timely information, and coming to a consensus between the patient, family and doctor, it might be possible to prevent many problems that arise when people make decisions about various interventions at the end of life. The family often become involved when deciding e.g., about PEG: *“…a large number of patients cannot decide for themselves… Then it is really about the family*, *the family decides…”* (HSCP). Medical staff sometimes encounters situations in which family members push the doctor to perform interventions, even though the patient would probably not desire it.

Support for family members who accompany the patient until the end of life is not less important. The family should receive some support even when deciding on the place of care at the end of life. Some family members described a problem with accepting the idea of putting their relative in an institution in the last stages.

Insufficient support for the family was also found out in the area of the preparation for the patient’s death. Adequate preparation can facilitate the process of mourning when the client dies: *“Not all families are ready for that… and you can see that they freeze*, *they can’t enter the room (when the patient dies)… they simply don’t want to cross the threshold*, *but*, *at the same time*, *they want to say good bye*, *but they are not ready for it*.*”*(HSCP).

## Discussion

Three categories were identified (family situation, role of the caregiver, and professional help), according to which we organized the unmet needs. Unmet needs did not differ significantly for individual diseases, but rather in the progress of the disease and the life stage of patients. When patients were of productive age, the burden arising from the disease was greater, even for family members. The unmet needs detailed in the study could be addressed by the introduction of the concept of neuropalliative and rehabilitative care.

Our results show that the care for a patient with PND significantly changes the life of a family caregiver, who is most often the partner of the ill person, or their daughter (or son or daughter-in-law). It places psychological demands on family members, affects their physical state, and impacts on their social relationships. Other authors also discovered significant changes in the lives of the caring family when they looked after patients with PD [36–38]. The caregivers of patients with PD may face a range of challenges. They might feel that “they have already lost a close relative” and experience a period of sadness. Moreover, the caregivers facing the high demands of care over the long term may have feelings of emptiness and guilt [39], which also proved the case in our research.

Caring for people with PND may result in a range of other negative consequences, including increased anxiety and depression, stress, exhaustion, social isolation, and fear of the future [40]. The level of psychological stress on family caregivers is a very significant phenomenon which should receive greater interest and attention from experts. Some of the consequences of long-term stress on family caregivers frequently include an illness or inability to continue providing the care. The preceding studies suggest that the level of caregiver stress is related to the length of providing care, and the health state of the patient, including the patient’s deteriorating disabilities and tendency to fall, along with the presence of psychiatric symptoms, including behavior disorders (like impulse-control disorder and apathy) [41,42]. Patients with PND in advanced stages may encounter all of these problems. The specific ways of coping with tasks and solving problems that the families embrace are affected by their system of convictions, and opinions of the individual family members and the family as a whole. The ability of the family to adapt to stress, to seek, and find balance under new conditions are quite crucial [43].

Our research revealed that when family members are adapting to the disease, various misunderstandings or conflicts arise, caused by differing views on the seriousness of the disease between the patient and the caregiver, problems coping with the patient’s dependence on the caregiver, and the interconnection of the physical and psychological state of the patient, typical in PND. Some participants expressed fears over whether they would be able to provide care for their relative over a long period. As other researchers have detailed, deep changes in the relationships between family members may occur [11], including the relationship between spouses, and between parents and children. Primarily, the level of dependence of the sick person on his or her spouse determines the changes in the relationship. At the same time, responsibilities are shifted. The caregiver may being to take care of financial issues, household duties, and may even have to leave work [44]. According to McLaughlin et al. [37], due to limited daily contact with the environment and the difficulties of care, the caregiver may become socially isolated. Other significant variables impacting on the caregiver’s quality of life are the patient’s ability to move, and cognitive function disorder, which is likely to appear in the later stages of many neurological diseases [45,46].

A another crucial discovery of our research is the lack of information in family members regarding the disease, care, and support, which makes the mutual understanding between the patient and the caring family more difficult, and increases the demands of care. Information provided by the doctor about these diseases is, nevertheless, regarded as a very important part of the support for the family [47]. Clear communication between the caregivers and medical staff is necessary to cope with the disease and the caring for the patient [7, 19, 48]. In the context of neuropalliative care, this should also include some discussion between the patient, family, and medical staff about the plan of the care, which, however, is usually not provided to family members in the Czech environment. Some studies indicate that such discussion might decrease the amount of misunderstandings in the family [49]. The cultural context may impact on the way autonomy is perceived and whether the ill person plays a role in deciding about the end of their life, the support of the palliative team, and family members [49, 50].

Support of family caregivers is important throughout the whole process of the disease, not only at the end of life. In neuropalliative and rehabilitative care problems should be identified and addressed at every stage of the disease. The support for family caregivers provided by the neurologist begins with an adequate assessment of their needs. Such an assessment should focus both on their personal wellbeing, as well as their ability to provide appropriate care for the patient [51]. There is evidence that evaluating caregivers’ needs and providing support significantly lowers the stress of the caregivers and the negative effects of long-term care [45, 52–54].

Those with PND and their caregivers are often scared of the dying process of dying and may fear dying more rather than death itself. According to The Irish Palliative Care in Parkinson’s Disease Group [39], it is necessary to inform people of what can be expected at the end of life; the people at the same time, they should have an opportunity to share their fears and anxieties. The caregivers should also be informed about the significant changes in providing care, which may be related to e.g., feeding and hydration. Support for caregivers is therefore an indispensable part of any palliative care, and may lower the risk of emotional exhaustion and burnout to a significant degree [7,55]. According to The Irish Palliative Care in Parkinson’s Disease Group [39], above all, support should be offered to caregivers on an individual basis, since, in some cases, group support can actually increase the anxiety of the caregivers (e.g., when they encounter those in a more advanced stage of the disease than their relative is at). Families could thus benefit from three levels of support: 1) general support and information (e.g., information on the process of mourning, help with practical tasks, social support), 2) further support (e.g., religious groups, community groups), 3) supportive therapy (e.g., psychologists, counsellors, doctors). Family caregivers should receive help primarily from social and health services. Our research revealed that, for the insignificant number of family members who are interested in them, such services are hard to obtain or even unavailable. The lack of information about these services also plays a negative role.

Palliative Care works through a multidisciplinary approach, by which patients’ problems, as well as those of family members, can be solved as they arise throughout the course of the disease, during the process of dying, and in the period of mourning.

### Study limitations

A limitation of the study may be the inclusion of patients one year after diagnosis, in order to focus the research on patients in an advanced stage of disease. The period of diagnosis is usually difficult for both patients and their family members, and is a topic that deserves further attention and research in itself. Another limitation may be the inclusion in the research sample of patients with multiple types of PND—diseases with very similar symptoms, but which differ in the time of onset and length of progression of the disease. For these reasons, each disease can have a specific impact on both patients and their caregivers. However, the description of unmet needs in several groups of PND patients may be sufficient to set up an appropriate health and social care system that is generally consistent for a variety of diseases. It is also specific to examine the unmet needs of family caregivers from the point of view of family members, as well as patients, and healthcare professionals. We believe that this perspective may extend the description of unmet needs, and thus facilitate the provision of adequate health and social care services.

## Conclusion

The response of family members revealed that when caring for an ill person with PND, lack of information about the disease represents a common problem, which complicates mutual understanding between the patient and the caring family, and increases caregivers’ stress. Family members expect health workers to provide them with support, including informing them about the possibilities of help available in the system of health and social care. In the Czech Republic, it is imperative to improve access to available, sustainable, and quality health and social services, not only for people with PND, but also for their informal caregivers. The services should be easily accessible to their users, which might prevent the social isolation or segregation of the users. Preventive measures may stop the complications of excessive psychological stress for family members when caring for a sick relative with PND right from the outset.
